# Characterizing the Cortical Oscillatory Response to TMS Pulse

**DOI:** 10.3389/fncel.2017.00038

**Published:** 2017-02-27

**Authors:** Maria Concetta Pellicciari, Domenica Veniero, Carlo Miniussi

**Affiliations:** ^1^Cognitive Neuroscience Section, IRCCS Centro San Giovanni di Dio FatebenefratelliBrescia, Italy; ^2^Centre for Cognitive Neuroimaging, Institute of Neuroscience and Psychology, University of GlasgowGlasgow, UK; ^3^Center for Mind/Brain Sciences - CIMeC, University of TrentoRovereto, Italy

**Keywords:** transcranial magnetic stimulation, TMS-EEG, cortical oscillatory activity, time-frequency representation, evoked, induced, total oscillatory response

In recent years, various techniques have been adopted to study brain functions *in vivo*. In this context, the combination of transcranial magnetic stimulation with electroencephalography (TMS-EEG) represents a powerful tool for investigating brain states and their dynamics. TMS-EEG allows the measurement of cortical reactivity, effective connectivity and dynamic properties of a given cortical area or network (Komssi and Kähkönen, 2006; Miniussi and Thut, 2010; Rogasch and Fitzgerald, 2013; Bortoletto et al., 2015). Specifically, the combination of TMS and EEG to study oscillatory cortical activity has proven to be a valuable technique to characterize the oscillatory activity of an area (Rosanova et al., 2009), to discriminate between normal and clinical cortical oscillatory patterns (Ferrarelli et al., 2008; Canali et al., 2015; Pellicciari et al., 2016, 2017), to investigate changes caused by experimental manipulative approaches (Veniero et al., 2013; Casula et al., 2016) and to evaluate the causal role of specific oscillatory network activity during task execution (Picazio et al., 2014).

Regarding the methods used to examine the brain dynamics triggered by a TMS pulse, many studies have adopted a time-frequency representation (TFR) approach. At a general level, TFR entails the spectral decomposition of the EEG signal resulting in a matrix expressing oscillatory power as a function of time and frequency (e.g., wavelet transforms, Hilbert transform, short-term Fourier analysis). In order to extract the frequency and amplitude of cortical oscillations over time (i.e., the value used for the TFR), two approaches are generally used: the first one is focused on evoked oscillatory response (EOR), while the second one accounts for the so-called induced oscillatory response (IOR). The latter is actually better characterized by the definition of total oscillatory response (TOR) (Roach and Mathalon, 2008; Herrmann et al., 2014). In a specific manner, EOR represents the averaged activity across trials, both time- and phase-locked (i.e., evoked) to the event onset, and this equates to the spectral decomposition of event-related potentials (Mouraux and Iannetti, 2008). Meanwhile, TOR implies that the time-frequency decomposition is performed for each single trial and then averaged. This approach captures phase-locked EOR but also non-phase-locked IOR activity in response to the stimulus onset. Consequently, to isolate the IOR, the TFR of phase-locked components EOR have to be removed from the total activity (TOR; Herrmann et al., 2014).

Considering that TMS-EEG is a rapidly growing tool to study oscillatory cortical activity, the aim of this work is to discuss what are the elements that we should consider relatively to TFR approaches, i.e., EOR and TOR triggered by a single TMS pulse. The idea is to promote a more justified use of these analyses and an accurate definition of the methodology used as well as the theoretical hypothesis underlying such use.

Regarding the EOR approach, the underlying rationale is that, by first averaging epochs in the time-frequency domain, the signal-to-noise ratio of EEG responses that are strictly related to the stimulus, i.e., time- and phase-locked to the TMS pulse, are increased. The results will show EEG changes that are considered to reflect systematic brain response. Therefore, the oscillatory activity occurring at a consistent latency and phase will survive the averaging process and will be seen in the TFR. Oscillations occurring after the stimulation with a varying time and/or phase jitter will be canceled out toward zero (Sauseng and Klimesch, 2008; Herrmann et al., 2014), assuming that non-phase locked signals represent uncorrelated noise in respect to the event of interest, i.e., the single TMS pulse. According to this framework and in analogy with sensory stimuli (Klimesch et al., 2007), when the TMS-EEG approach is used to evaluate the oscillatory activity evoked by single TMS pulse, the TMS affects brain activity through a transient phase alignment of the ongoing oscillations (phase-reset; Thut et al., 2011; Kawasaki et al., 2014), synchronizing neurons to fire at a specific frequency range, depending on the stimulated area (Van Der Werf and Paus, 2006; Rosanova et al., 2009; Herring et al., 2015). Therefore, TMS-triggered oscillations evaluated using an EOR approach should reflect physiological activity that is transiently revealed by the TMS pulse. However, one important point would be to understand whether a single TMS pulse is imposing an artificial activity or is instead acting by enhancing “naturally occurring” oscillations. Thut et al. (2011) indirectly addressed this issue and demonstrated that TMS-EORs depend on pre-stimulus activity, whereas more recently, Herring et al. (2015) showed that TMS oscillatory activity is generated by the same neurophysiological generator as spontaneous oscillations. Taken together, this evidence supports the idea that TMS-EOR is a valuable measure for studying the causal role of neuronal oscillations characterizing a given area.

Nevertheless, although a single TMS pulse synchronizes pre-existing and ongoing oscillations rather than eliciting and inducing new neural responses (Van Der Werf and Paus, 2006; Thut et al., 2011), effects on frequencies, other than the evoked ones, cannot be excluded. Moreover, it remains to be clarified whether TMS mainly enhances the “natural” frequency of the target area, allowing the cortical response to a TMS pulse to be considered a “stereotyped” oscillation (Veniero et al., 2011), or whether this activity is also significantly related to the subject's state. The latter case should be defined as a natural-state-dependent frequency triggered by TMS pulse. To disentangle whether and to what extent the cortical oscillatory activity related to TMS pulse can be considered specific to the stimulated cortex (Rosanova et al., 2009) or whether it is more appropriately scribed to a given state, we should compare different TFR approaches (EOR vs. TOR). Such comparison should be performed in different subject's state (i.e., in rest or during a task, in wakefulness or sleep) considering with attention the contribute of baseline activity. The baseline issue, i.e., the EEG activity before each single TMS pulse, will not be discussed here, even if it is a very relevant methodological and theoretical topic (e.g., Morcom and Fletcher, 2007).

The TOR approach could be able to capture a more complex cortical oscillatory response to TMS, comprising both EOR and IOR. In this context, to obtain the TFR, time-frequency analysis locked to the TMS pulse is performed on each single trial and then averaged. Therefore, TOR captures not only the time- and phase-locked response to TMS pulse (i.e., EOR) but also the brain activity defined as non-stationary, including time-locked responses with jittered latency across trials (Tallon-Baudry and Bertrand, 1999; Mutanen et al., 2013) and not necessarily phase-locked to the TMS pulse (i.e., IOR). At a more general level, the TOR procedure enhances the signal-to-noise ratio of both phase-locked and non-phase-locked event-related EEG responses, thereby allowing the description of possible event-related transient modulations of oscillatory activity (Mouraux and Iannetti, 2008). Note that to obtain the IOR, the EOR must be removed from the single-trial-based estimate, subtracting it from TOR (Roach and Mathalon, 2008; Herrmann et al., 2014). However, in many studies, the TOR is erroneously referred to as the induced response, and it is not clearly stated whether any subtraction has been performed (see Figure 1). For example, in a study by Bergmann et al. (2012), TMS-triggered oscillations were used to evaluate the state-dependent shift in neocortical excitability during different phases of a neuronal oscillation. In this work, it would have been interesting to discriminate the specific contribute of EOR and IOR, whereas the authors reported only the TOR, but refer to it as IOR. Nevertheless, of particular interest in this work is the idea of subtracting the no-TMS trials from averages derived from TMS trials to identify the brain activity generated by the TMS pulse (after removing any oscillatory activity associated with the endogenous activity).

**Figure 1:**
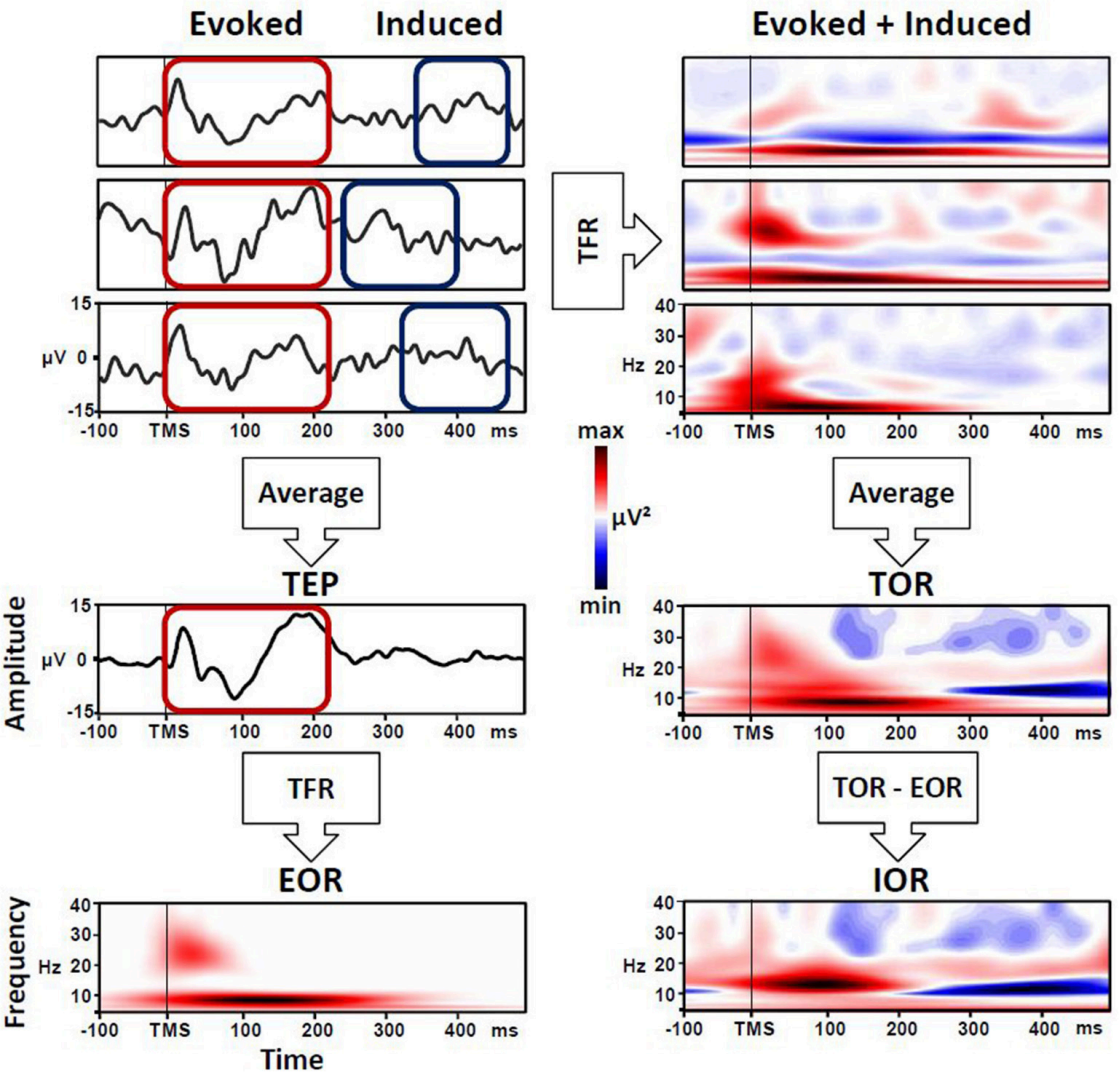
**Evoked (EOR), induced (IOR) and total oscillatory response (TOR) triggered by TMS pulse. Left panel:** EOR is the time-frequency representation (TFR) of the average across all single cortical responses to TMS pulse (TEP). **Right panel:** TOR is the average of the TFR of each single response to TMS pulse, and includes EOR and IOR. To isolate pure IOR, the EOR must be removed from the TOR.

If we simplistically consider that the synchronous activation of a large number of neurons by means of a magnetic pulse will likely cause an immediate phase reset, therefore, the EOR might represent an ideal measure. In this context, if we are interested in excitability changes, the EOR can detect them, highlighting the difference in terms of oscillatory power in the immediate response to TMS. However, using the EOR approach might not be justifiable if we bear in mind that the brain is a nonlinear system generating non-stationary signals that might not necessarily be time-locked with the event (Mutanen et al., 2013). Moreover, we must consider that even a single TMS pulse can generate a complex cascade of events characterized by a certain level of variability, in terms of phase and latency. A clear example of this point comes from a study by Moliadze et al. (2003) in which they demonstrated that depending on the stimulation intensity, a single magnetic pulse generates alternating episodes of facilitation and suppression as indexed by the number of neuronal spikes. Interestingly, the duration of the facilitation and in particular of the suppression, which last up to a few seconds under some conditions, seem compatible with the idea that in addition to responses immediately generated by a phase reset, TMS might also trigger additional subsequent endogenous oscillations mediated by metabotropic receptors or by second-messenger systems, which would not be time-locked with TMS. We speculate that these late and non-stationary effects might be related and might predict TMS after-effects (Veniero et al., 2015). In this framework, TOR or IOR may be the best approaches for highlighting additional oscillatory responses triggered by TMS.

Therefore, regardless of whether evoked or total oscillatory activity is analyzed, it is worth noting that one should consider the contribution of several factors that could affect TMS-triggered oscillatory activity. We know that different neuronal states determine the reactivity of the cortex to TMS (Silvanto et al., 2007; Paulus and Rothwell, 2016), therefore it is likely that the relevance of one analysis method or the other (EOR vs. TOR) depends on the context. For example, it has been suggested that in the resting state (e.g., no sensory input), the TMS pulse mainly acts as a phase reset, causing a precise frequency oscillation (Moliadze et al., 2003), whereas stimulating the same area during a specific activity might produce a more complex pattern due to specific interactions between TMS and the brain state. Therefore, the cortical response in terms of oscillatory activity can change depending on the subject state (rest vs. activity), and the EOR and TOR approaches, with their different theoretical frameworks, can represent the two situations in different lights.

Additionally, several single TMS pulses could represent *per se* a manipulative event, able to induce neuronal activity changes (Allen et al., 2007; Funke and Benali, 2010) by means of additive and cumulative mechanisms, and thereby influencing the background activity of the stimulated region (Stamoulis et al., 2011; Fedele et al., 2016; Pellicciari et al., 2016). Consequently, when applying several TMS pulses, a complex additive interaction between the exogenous event and endogenous neuronal cortical excitability is triggered (Kawasaki et al., 2014). Therefore, it is important to understand whether and how single TMS pulse *per se* affects the neuronal oscillations, and additionally in which terms: “phase-reset” of ongoing oscillations or “added-energy.” Then if TMS adds energy to the system, it should be evaluated if this energy is expressed in terms of oscillatory power increase of a specific frequency or is a temporal modulation of cortical oscillations in others frequency bands (Formaggio et al., 2016). In other words, it is critical to establish whether a TMS protocol applied to probe the brain state is actually changing the brain response by itself. This would allow better evaluation of the impact of a manipulative approach (e.g., repetitive TMS) itself vs. the testing approach (e.g., single TMS pulse).

The full potential of TMS-EEG for studying the oscillatory cortical activity depends strongly on the understanding of possible neural effects of single TMS pulses on baseline cortical activity and on the best method to adequately measure the cortical oscillatory responses triggered by TMS. Given that the complex interaction between TMS pulse and baseline oscillatory activity is probably detected in different manners by EOR and TOR approaches, we should decide and precisely describe, based on the experimental hypothesis, why to use one approach or the other for the analysis of TMS triggered oscillations.

## Funding

This work was supported by grants of the Italian Ministry of Health: GR-2011-02349998 to MP and RF-2013-02356444 to CM.

## Author contributions

MP, DV, and CM have equally contributed to the manuscript.

### Conflict of interest statement

The authors declare that the research was conducted in the absence of any commercial or financial relationships that could be construed as a potential conflict of interest.
